# 2-Amino-5-methyl­pyridinium 3-carb­oxy-4-hy­droxy­benzene­sulfonate

**DOI:** 10.1107/S1600536810029636

**Published:** 2010-07-31

**Authors:** Madhukar Hemamalini, Hoong-Kun Fun

**Affiliations:** a X-ray Crystallography Unit, School of Physics, Universiti Sains Malaysia, 11800 USM, Penang, Malaysia

## Abstract

The asymmetric unit of the title salt, C_6_H_9_N_2_^+^·C_7_H_5_O_6_S^−^, contains two crystallographically independent 2-amino-5-methylpyridinium cations and two sulfosalicylate anions. In the crystal structure, the sulfonate group of each 3-carb­oxy-4-hy­droxy­benzene­sulfonate anion inter­acts with the corresponding 2-amino-5-methyl­pyridinium cation *via* a pair of N—H⋯O hydrogen bonds, forming an *R*_2_^2^(8) ring motif. The ionic units are linked by N—H⋯O, O—H⋯O and C—H⋯O hydrogen bonds. Furthermore, the crystal structure is stabilized by π–π inter­actions between the benzene and pyridine rings [centroid–centroid distances = 3.5579 (8) and 3.8309 (8) Å]. There are also intra­molecular O—H⋯O hydrogen bonds in the anions, which generate *S*(6) ring motifs.

## Related literature

For details of weak inter­actions, see: Moghimi *et al.* (2002 ▶); Aghabozorg *et al.* (2005 ▶). For applications of sulfosalicylic acid, see: Smith *et al.* (2004 ▶); Raj *et al.* (2003 ▶); Muthiah *et al.* (2003 ▶); Wang & Wei (2007 ▶). For related structures, see: Nahringbauer & Kvick (1977 ▶). For hydrogen-bond motifs, see: Bernstein *et al.* (1995 ▶). For bond-length data, see: Allen *et al.* (1987 ▶). For the stability of the temperature controller used in the data collection, see: Cosier & Glazer (1986 ▶).
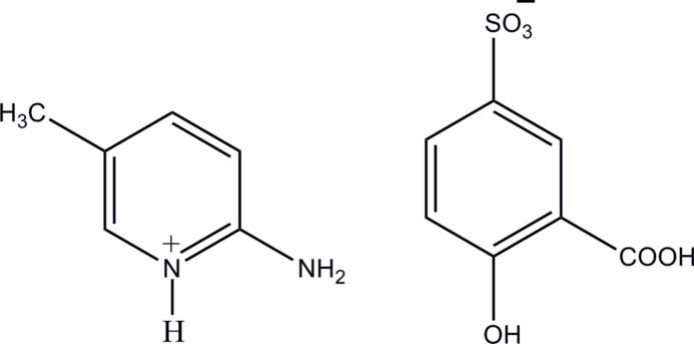


## Experimental

### 

#### Crystal data

C_6_H_9_N_2_^+^·C_7_H_5_O_6_S^−^
*M* *_r_* = 326.32
Triclinic, 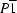

*a* = 7.8635 (1) Å
*b* = 10.8827 (1) Å
*c* = 16.3907 (2) Å
α = 84.612 (1)°
β = 81.802 (1)°
γ = 86.290 (1)°
*V* = 1380.31 (3) Å^3^
*Z* = 4
Mo *K*α radiation
μ = 0.27 mm^−1^
*T* = 100 K
0.27 × 0.16 × 0.15 mm

#### Data collection

Bruker APEXII CCD area-detector diffractometer
Absorption correction: multi-scan (*SADABS*; Bruker, 2009 ▶) *T*_min_ = 0.931, *T*_max_ = 0.960
28351 measured reflections
7325 independent reflections
6209 reflections with *I* > 2σ(*I*)
*R*_int_ = 0.027

#### Refinement

*R*[*F* ^2^ > 2σ(*F* ^2^)] = 0.034
*wR*(*F* ^2^) = 0.092
*S* = 1.04
7325 reflections
439 parameters
H atoms treated by a mixture of independent and constrained refinement
Δρ_max_ = 0.45 e Å^−3^
Δρ_min_ = −0.42 e Å^−3^

### 

Data collection: *APEX2* (Bruker, 2009 ▶); cell refinement: *SAINT* (Bruker, 2009 ▶); data reduction: *SAINT*; program(s) used to solve structure: *SHELXTL* (Sheldrick, 2008 ▶); program(s) used to refine structure: *SHELXTL*; molecular graphics: *SHELXTL*; software used to prepare material for publication: *SHELXTL* and *PLATON* (Spek, 2009 ▶).

## Supplementary Material

Crystal structure: contains datablocks global, I. DOI: 10.1107/S1600536810029636/is2580sup1.cif

Structure factors: contains datablocks I. DOI: 10.1107/S1600536810029636/is2580Isup2.hkl

Additional supplementary materials:  crystallographic information; 3D view; checkCIF report

## Figures and Tables

**Table 1:** Hydrogen-bond geometry (Å, °)

*D*—H⋯*A*	*D*—H	H⋯*A*	*D*⋯*A*	*D*—H⋯*A*
O4*A*—H1*OA*⋯O6*A*	0.88 (2)	1.84 (2)	2.6135 (14)	147 (2)
O4*A*—H1*OA*⋯O1*B*^i^	0.88 (2)	2.39 (2)	2.9581 (14)	123.2 (18)
O5*A*—H2*OA*⋯O2*B*^ii^	0.86 (2)	1.80 (2)	2.6609 (14)	172 (2)
O4*B*—H1*OB*⋯O5*B*	0.86 (3)	1.83 (2)	2.5918 (14)	147 (2)
O4*B*—H1*OB*⋯O2*A*^iii^	0.86 (3)	2.45 (2)	3.0349 (14)	125.4 (18)
O6*B*—H2*OB*⋯O1*A*	0.86 (2)	1.81 (2)	2.6664 (14)	178 (2)
N1*A*—H1*NA*⋯O3*A*^iv^	0.894 (19)	2.066 (19)	2.9057 (15)	156.0 (17)
N2*A*—H2*NA*⋯O2*A*^iv^	0.878 (19)	2.167 (19)	3.0043 (16)	159.1 (17)
N2*A*—H2*NA*⋯O5*B*^v^	0.878 (19)	2.417 (19)	2.8235 (16)	108.7 (13)
N2*A*—H3*NA*⋯O1*A*^v^	0.88 (2)	2.17 (2)	3.0472 (16)	175.6 (15)
N1*B*—H1*NB*⋯O3*B*^ii^	0.87 (2)	2.02 (2)	2.8547 (16)	161 (2)
N2*B*—H2*NB*⋯O1*B*^ii^	0.90 (2)	2.04 (2)	2.9188 (17)	166 (2)
N2*B*—H2*NB*⋯O6*A*^vi^	0.90 (2)	2.45 (2)	2.8254 (16)	105.9 (17)
N2*B*—H3*NB*⋯O2*B*^i^	0.87 (2)	2.26 (2)	3.1270 (17)	177.1 (18)
C7*A*—H7*AA*⋯O4*B*^iii^	0.93	2.58	3.4257 (16)	152
C7*B*—H7*BA*⋯O4*A*^i^	0.93	2.48	3.3116 (16)	148

Symmetry codes: (i) 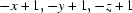; (ii) 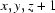; (iii) 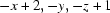; (iv) 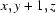; (v) 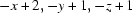; (vi) 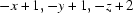.

**Fig. 1.**
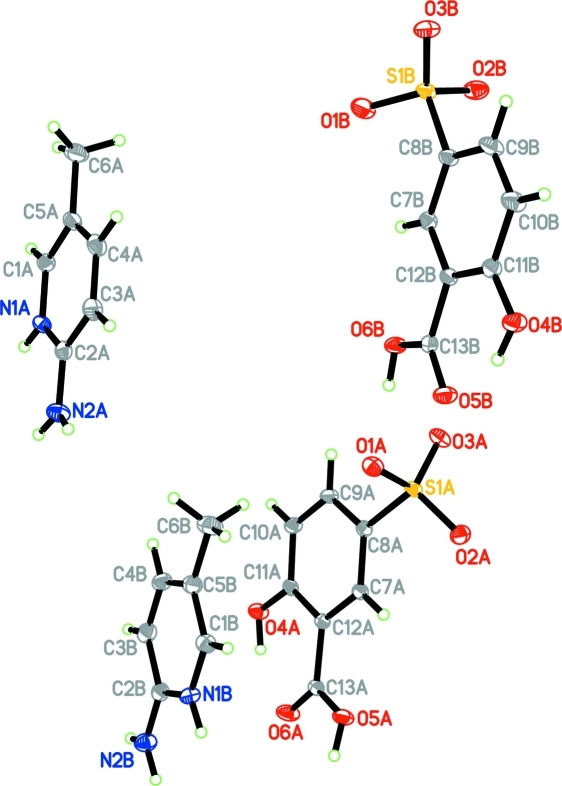
The asymmetric unit of the title compound. Displacement ellipsoids are drawn at the 50% probability level.

**Fig. 2.**
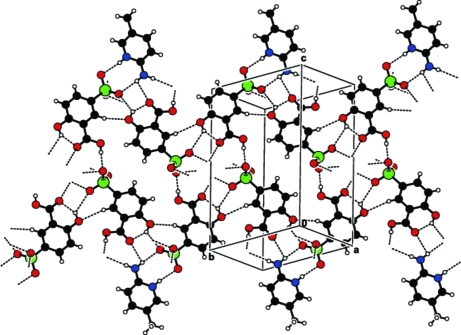
Hydrogen bonding patterns in compound (I).

**Fig. 3.**
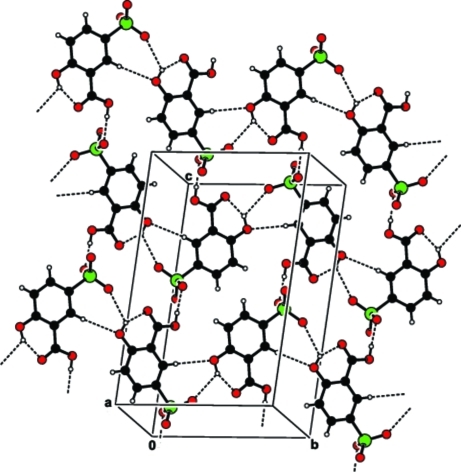
Supramolecular sheet made up of 3-carboxy-4-hydroxybenzenesulfonate anions. .

## supplementary crystallographic information

### Comment

Weak interactions, such as hydrogen bonding and π–π stacking, have attracted much interest as a result of their significance in chemistry and biology, especially in the field of crystal engineering (Moghimi *et al.*, 2002; Aghabozorg *et al.*, 2005). 5-Sulfosalicylic acid (3-carboxy-4-hydroxybenzenesulfonic acid), is a particularly strong organic acid which is capable of protonating *N*-containing heterocycles and other Lewis bases (Smith *et al.*; 2004, Raj *et al.*, 2003; Muthiah *et al.*, 2003; Wang & Wei, 2007).  As part of our research programme aiming to gain further insight into hydrogen-bonding interactions involving 2-amino-5-methylpyridine and 3-carboxy-4-hydroxybenzenesulfonic acid, the present work has been undertaken.

The asymmetric unit of the title salt consists of two crystallographically independent 2-amino-5-methylpyridinium cations (A & B) and two sulfosalicylate anions (A & B) (Fig. 1). Each 2-amino-5-methylpyridinium cation is planar, with a maximum deviation of 0.003 (1) Å for C5A atom (molecule A) and 0.008 (1) Å for atom C2B (molecule B).  In the cations, protonation at atoms N1A and N1B lead to slight increases in the C1A—N1A—C2A [123.30 (12)°] and C1B—N1B—C2B [123.07 (12)°] angles compared to those observed in an unprotonated structure (Nahringbauer & Kvick, 1977). The bond lengths (Allen *et al.*, 1987) and angles are normal.

In the crystal structure (Fig. 2), the sulfonate group of each 3-carboxy-4- hydroxybenzenesulfonate anion interacts with the corresponding 2-amino- 5-methylpyridinium cation *via* a pair of N—H···O hydrogen bonds, forming an *R*_2_^2^(8) ring motif (Bernstein *et al.*, 1995).  The ionic units are linked by N—H···O and O—H···O (Table 1) hydrogen bonds. The 3-carboxy 4-hydroxybenzenesulfonate anions self-assemble *via* O—H···O and C—H···O interactions, leading to the formation of a sheet-like structure, as shown in Fig. 3. There are intramolecular hydrogen bonds between the -OH and -COOH groups in sulfosalicylate anions, which generate *S*(6) ring motifs. The crystal structure is further stabilized by π–π interactions between the cations and anions [centroid-to-centroid distance = 3.5579 (8) Å (1-x, 1-y, 1-z) and 3.8309 (8) Å (2-x, 1-y, 1-z)].

### Experimental

A hot methanol solution (20 ml) of 2-amino-5-methylpyridine (27 mg, Aldrich) and sulfosalicylic acid (54 mg, Merck) were mixed and warmed over a heating magnetic stirrer hotplate for a few minutes. The resulting solution was allowed to cool slowly at room temperature and crystals of the title compound appeared after a few days.

### Refinement

Atoms H1OA, H2OA, H1OB, H2OB, H1NA, H2NA, H3NA, H1NB, H2NB and H3NB were located in a difference Fourier map and were refined freely [N—H = 0.87 (2)–0.90 (2) Å and O—H = 0.86 (2)–0.88 (2) Å]. The remaining hydrogen atoms were positioned geometrically [C—H = 0.93 or 0.96 Å] and were refined using a riding model, with *U*_iso_(H) = 1.2 or 1.5*U*_eq_(C).

### Crystal data

**Table:**

C_6_H_9_N_2_^+^·C_7_H_5_O_6_S^−^	*Z* = 4
*M**_r_* = 326.32	*F*(000) = 680
Triclinic, *P*1	*D*_x_ = 1.570 Mg m^−^^3^
Hall symbol: -P 1	Mo *K*α radiation, λ = 0.71073 Å
*a* = 7.8635 (1) Å	Cell parameters from 9890 reflections
*b* = 10.8827 (1) Å	θ = 2.4–30.2°
*c* = 16.3907 (2) Å	µ = 0.27 mm^−^^1^
α = 84.612 (1)°	*T* = 100 K
β = 81.802 (1)°	Block, colourless
γ = 86.290 (1)°	0.27 × 0.16 × 0.15 mm
*V* = 1380.31 (3) Å^3^	

### Data collection

**Table:**

Bruker APEXII CCD area-detector diffractometer	7325 independent reflections
Radiation source: fine-focus sealed tube	6209 reflections with *I* > 2σ(*I*)
graphite	*R*_int_ = 0.027
φ and ω scans	θ_max_ = 29.0°, θ_min_ = 1.3°
Absorption correction: multi-scan (*SADABS*; Bruker, 2009)	*h* = −10→10
*T*_min_ = 0.931, *T*_max_ = 0.960	*k* = −14→14
28351 measured reflections	*l* = −22→22

### Refinement

**Table:**

Refinement on *F*^2^	Primary atom site location: structure-invariant direct methods
Least-squares matrix: full	Secondary atom site location: difference Fourier map
*R*[*F*^2^ > 2σ(*F*^2^)] = 0.034	Hydrogen site location: inferred from neighbouring sites
*wR*(*F*^2^) = 0.092	H atoms treated by a mixture of independent and constrained refinement
*S* = 1.04	*w* = 1/[σ^2^(*F*_o_^2^) + (0.0439*P*)^2^ + 0.7462*P*] where *P* = (*F*_o_^2^ + 2*F*_c_^2^)/3
7325 reflections	(Δ/σ)_max_ = 0.001
439 parameters	Δρ_max_ = 0.45 e Å^−^^3^
0 restraints	Δρ_min_ = −0.42 e Å^−^^3^

### Special details

**Table:**

Experimental. The crystal was placed in the cold stream of an Oxford Cryosystems Cobra open-flow nitrogen cryostat (Cosier & Glazer, 1986) operating at 100.0 (1) K.
Geometry. All esds (except the esd in the dihedral angle between two l.s. planes) are estimated using the full covariance matrix. The cell esds are taken into account individually in the estimation of esds in distances, angles and torsion angles; correlations between esds in cell parameters are only used when they are defined by crystal symmetry. An approximate (isotropic) treatment of cell esds is used for estimating esds involving l.s. planes.
Refinement. Refinement of F^2^ against ALL reflections. The weighted R-factor wR and goodness of fit S are based on F^2^, conventional R-factors R are based on F, with F set to zero for negative F^2^. The threshold expression of F^2^ > 2sigma(F^2^) is used only for calculating R-factors(gt) etc. and is not relevant to the choice of reflections for refinement. R-factors based on F^2^ are statistically about twice as large as those based on F, and R- factors based on ALL data will be even larger.

### Fractional atomic coordinates and isotropic or equivalent isotropic displacement parameters (Å^2^)

**Table:**

	*x*	*y*	*z*	*U*_iso_*/*U*_eq_	
N1A	0.70541 (15)	0.97702 (11)	0.37753 (7)	0.0143 (2)	
N2A	0.87358 (16)	0.87759 (12)	0.47109 (8)	0.0187 (2)	
C1A	0.65985 (18)	1.00244 (13)	0.30014 (8)	0.0157 (3)	
H1AA	0.5659	1.0567	0.2928	0.019*	
C2A	0.83866 (17)	0.89880 (12)	0.39405 (8)	0.0148 (3)	
C3A	0.93427 (18)	0.84175 (13)	0.32623 (9)	0.0184 (3)	
H3AA	1.0272	0.7871	0.3347	0.022*	
C4A	0.88974 (19)	0.86717 (13)	0.24861 (9)	0.0196 (3)	
H4AA	0.9532	0.8292	0.2047	0.024*	
C5A	0.74866 (18)	0.95025 (13)	0.23365 (9)	0.0177 (3)	
C6A	0.6994 (2)	0.97934 (16)	0.14844 (9)	0.0254 (3)	
H6AA	0.6016	1.0371	0.1508	0.038*	
H6AB	0.6711	0.9048	0.1276	0.038*	
H6AC	0.7941	1.0148	0.1124	0.038*	
N1B	0.80370 (15)	0.51730 (11)	0.82703 (7)	0.0164 (2)	
N2B	0.62446 (18)	0.60847 (12)	0.93174 (8)	0.0203 (3)	
C1B	0.85961 (18)	0.50127 (13)	0.74565 (8)	0.0171 (3)	
H1BA	0.9540	0.4474	0.7322	0.021*	
C2B	0.66881 (18)	0.59477 (12)	0.85203 (9)	0.0163 (3)	
C3B	0.58139 (18)	0.65888 (13)	0.78921 (9)	0.0185 (3)	
H3BA	0.4866	0.7120	0.8036	0.022*	
C4B	0.63620 (19)	0.64269 (13)	0.70806 (9)	0.0195 (3)	
H4BA	0.5779	0.6852	0.6676	0.023*	
C5B	0.78034 (18)	0.56232 (13)	0.68388 (9)	0.0179 (3)	
C6B	0.8423 (2)	0.54427 (16)	0.59433 (9)	0.0256 (3)	
H6BA	0.9511	0.4983	0.5899	0.038*	
H6BB	0.8552	0.6234	0.5635	0.038*	
H6BC	0.7600	0.4996	0.5725	0.038*	
S1A	0.65009 (4)	0.18717 (3)	0.536127 (19)	0.01282 (8)	
O1A	0.79222 (12)	0.26353 (9)	0.49587 (6)	0.0174 (2)	
O2A	0.71260 (13)	0.07391 (9)	0.57916 (6)	0.0191 (2)	
O3A	0.53262 (13)	0.16622 (9)	0.47817 (6)	0.0181 (2)	
O4A	0.25189 (13)	0.48004 (9)	0.79013 (6)	0.0177 (2)	
O5A	0.64439 (13)	0.22501 (9)	0.85364 (6)	0.0190 (2)	
O6A	0.42614 (14)	0.35554 (10)	0.89784 (6)	0.0220 (2)	
C7A	0.56636 (17)	0.25282 (12)	0.69411 (8)	0.0127 (2)	
H7AA	0.6474	0.1912	0.7079	0.015*	
C8A	0.53508 (17)	0.27527 (12)	0.61292 (8)	0.0128 (2)	
C9A	0.41623 (17)	0.36980 (12)	0.59126 (8)	0.0146 (2)	
H9AA	0.3982	0.3861	0.5364	0.018*	
C10A	0.32577 (18)	0.43881 (12)	0.65118 (8)	0.0153 (3)	
H10A	0.2478	0.5021	0.6364	0.018*	
C11A	0.35090 (17)	0.41392 (12)	0.73440 (8)	0.0133 (2)	
C12A	0.47654 (17)	0.32248 (12)	0.75539 (8)	0.0127 (2)	
C13A	0.51185 (17)	0.30248 (12)	0.84203 (8)	0.0144 (2)	
S1B	0.85292 (4)	0.31409 (3)	0.00943 (2)	0.01532 (8)	
O1B	0.79350 (15)	0.43064 (10)	0.04277 (6)	0.0251 (2)	
O2B	0.70905 (13)	0.23442 (10)	0.00786 (6)	0.0218 (2)	
O3B	0.95916 (14)	0.32797 (10)	−0.07089 (6)	0.0210 (2)	
O4B	1.26682 (13)	0.03415 (9)	0.24475 (6)	0.0184 (2)	
O5B	1.06979 (13)	0.13883 (9)	0.36187 (6)	0.0189 (2)	
O6B	0.84902 (13)	0.26692 (9)	0.33138 (6)	0.0166 (2)	
C7B	0.93897 (17)	0.25027 (12)	0.16353 (8)	0.0133 (2)	
H7BA	0.8489	0.3052	0.1816	0.016*	
C8B	0.98051 (17)	0.23526 (12)	0.08005 (8)	0.0144 (2)	
C9B	1.11603 (19)	0.15321 (14)	0.05255 (9)	0.0199 (3)	
H9BA	1.1431	0.1435	−0.0037	0.024*	
C10B	1.21005 (19)	0.08647 (14)	0.10831 (9)	0.0197 (3)	
H10B	1.3000	0.0319	0.0895	0.024*	
C11B	1.17031 (17)	0.10077 (12)	0.19298 (8)	0.0146 (3)	
C12B	1.03250 (17)	0.18283 (12)	0.22094 (8)	0.0129 (2)	
C13B	0.98732 (17)	0.19430 (12)	0.31039 (8)	0.0135 (2)	
H1OA	0.281 (3)	0.455 (2)	0.8388 (14)	0.043 (6)*	
H2OA	0.656 (3)	0.225 (2)	0.9052 (14)	0.042 (6)*	
H1OB	1.228 (3)	0.051 (2)	0.2945 (16)	0.053 (7)*	
H2OB	0.833 (3)	0.264 (2)	0.3844 (15)	0.047 (6)*	
H1NA	0.640 (2)	1.0158 (17)	0.4175 (12)	0.025 (5)*	
H2NA	0.814 (2)	0.9185 (17)	0.5103 (12)	0.023 (5)*	
H3NA	0.970 (3)	0.8352 (17)	0.4780 (11)	0.022 (5)*	
H1NB	0.858 (3)	0.474 (2)	0.8631 (13)	0.036 (6)*	
H2NB	0.683 (3)	0.565 (2)	0.9689 (14)	0.040 (6)*	
H3NB	0.531 (3)	0.6531 (18)	0.9467 (12)	0.029 (5)*	

### Atomic displacement parameters (Å^2^)

**Table:**

	*U*^11^	*U*^22^	*U*^33^	*U*^12^	*U*^13^	*U*^23^
N1A	0.0140 (5)	0.0164 (5)	0.0126 (5)	0.0000 (4)	−0.0004 (4)	−0.0044 (4)
N2A	0.0164 (6)	0.0243 (6)	0.0155 (6)	0.0031 (5)	−0.0034 (5)	−0.0036 (5)
C1A	0.0158 (6)	0.0167 (6)	0.0152 (6)	−0.0020 (5)	−0.0031 (5)	−0.0015 (5)
C2A	0.0140 (6)	0.0143 (6)	0.0164 (6)	−0.0022 (5)	−0.0014 (5)	−0.0022 (5)
C3A	0.0165 (6)	0.0170 (6)	0.0213 (7)	0.0016 (5)	0.0001 (5)	−0.0050 (5)
C4A	0.0206 (7)	0.0199 (7)	0.0180 (7)	−0.0031 (5)	0.0035 (5)	−0.0078 (5)
C5A	0.0196 (7)	0.0196 (6)	0.0147 (6)	−0.0064 (5)	−0.0012 (5)	−0.0034 (5)
C6A	0.0289 (8)	0.0334 (8)	0.0148 (7)	−0.0055 (7)	−0.0023 (6)	−0.0044 (6)
N1B	0.0175 (6)	0.0181 (6)	0.0130 (5)	0.0004 (4)	−0.0030 (4)	0.0015 (4)
N2B	0.0216 (6)	0.0233 (6)	0.0149 (6)	0.0020 (5)	−0.0006 (5)	−0.0007 (5)
C1B	0.0164 (6)	0.0193 (6)	0.0155 (6)	−0.0016 (5)	−0.0005 (5)	−0.0023 (5)
C2B	0.0168 (6)	0.0153 (6)	0.0166 (6)	−0.0031 (5)	−0.0013 (5)	−0.0002 (5)
C3B	0.0161 (6)	0.0178 (6)	0.0213 (7)	0.0003 (5)	−0.0038 (5)	0.0006 (5)
C4B	0.0209 (7)	0.0190 (7)	0.0198 (7)	−0.0038 (5)	−0.0085 (6)	0.0033 (5)
C5B	0.0182 (7)	0.0213 (7)	0.0149 (6)	−0.0065 (5)	−0.0027 (5)	0.0001 (5)
C6B	0.0286 (8)	0.0335 (8)	0.0152 (7)	−0.0066 (7)	−0.0035 (6)	−0.0012 (6)
S1A	0.01335 (15)	0.01459 (15)	0.01021 (14)	0.00201 (11)	−0.00056 (11)	−0.00296 (11)
O1A	0.0158 (5)	0.0214 (5)	0.0144 (5)	−0.0015 (4)	0.0018 (4)	−0.0034 (4)
O2A	0.0237 (5)	0.0166 (5)	0.0157 (5)	0.0069 (4)	−0.0010 (4)	−0.0019 (4)
O3A	0.0169 (5)	0.0235 (5)	0.0151 (5)	0.0014 (4)	−0.0035 (4)	−0.0078 (4)
O4A	0.0200 (5)	0.0201 (5)	0.0125 (5)	0.0077 (4)	−0.0026 (4)	−0.0053 (4)
O5A	0.0229 (5)	0.0211 (5)	0.0143 (5)	0.0066 (4)	−0.0085 (4)	−0.0050 (4)
O6A	0.0271 (6)	0.0263 (5)	0.0125 (5)	0.0095 (4)	−0.0047 (4)	−0.0065 (4)
C7A	0.0124 (6)	0.0130 (6)	0.0131 (6)	0.0009 (5)	−0.0025 (5)	−0.0019 (4)
C8A	0.0132 (6)	0.0134 (6)	0.0114 (6)	0.0001 (5)	−0.0005 (5)	−0.0027 (4)
C9A	0.0162 (6)	0.0165 (6)	0.0111 (6)	0.0010 (5)	−0.0031 (5)	−0.0001 (5)
C10A	0.0161 (6)	0.0156 (6)	0.0141 (6)	0.0038 (5)	−0.0039 (5)	−0.0009 (5)
C11A	0.0136 (6)	0.0131 (6)	0.0133 (6)	0.0012 (5)	−0.0011 (5)	−0.0038 (5)
C12A	0.0142 (6)	0.0125 (6)	0.0118 (6)	−0.0002 (5)	−0.0030 (5)	−0.0017 (4)
C13A	0.0170 (6)	0.0132 (6)	0.0135 (6)	0.0006 (5)	−0.0043 (5)	−0.0024 (5)
S1B	0.01759 (16)	0.01824 (16)	0.01035 (15)	0.00332 (12)	−0.00421 (12)	−0.00176 (11)
O1B	0.0344 (6)	0.0232 (5)	0.0183 (5)	0.0122 (5)	−0.0093 (5)	−0.0059 (4)
O2B	0.0212 (5)	0.0286 (6)	0.0165 (5)	−0.0029 (4)	−0.0080 (4)	0.0019 (4)
O3B	0.0228 (5)	0.0264 (5)	0.0124 (5)	0.0021 (4)	−0.0017 (4)	0.0015 (4)
O4B	0.0186 (5)	0.0211 (5)	0.0150 (5)	0.0075 (4)	−0.0045 (4)	−0.0012 (4)
O5B	0.0220 (5)	0.0216 (5)	0.0127 (4)	0.0058 (4)	−0.0041 (4)	−0.0004 (4)
O6B	0.0187 (5)	0.0192 (5)	0.0107 (4)	0.0049 (4)	−0.0003 (4)	−0.0012 (4)
C7B	0.0128 (6)	0.0137 (6)	0.0136 (6)	0.0003 (5)	−0.0022 (5)	−0.0023 (5)
C8B	0.0153 (6)	0.0161 (6)	0.0122 (6)	0.0020 (5)	−0.0042 (5)	−0.0024 (5)
C9B	0.0221 (7)	0.0252 (7)	0.0125 (6)	0.0056 (6)	−0.0032 (5)	−0.0059 (5)
C10B	0.0193 (7)	0.0236 (7)	0.0155 (6)	0.0085 (6)	−0.0015 (5)	−0.0059 (5)
C11B	0.0141 (6)	0.0153 (6)	0.0145 (6)	0.0018 (5)	−0.0034 (5)	−0.0016 (5)
C12B	0.0135 (6)	0.0132 (6)	0.0125 (6)	−0.0003 (5)	−0.0023 (5)	−0.0021 (5)
C13B	0.0155 (6)	0.0123 (6)	0.0125 (6)	−0.0010 (5)	−0.0015 (5)	−0.0011 (4)

### Geometric parameters (Å, °)

**Table:**

N1A—C2A	1.3483 (18)	S1A—O1A	1.4771 (10)
N1A—C1A	1.3655 (17)	S1A—C8A	1.7639 (13)
N1A—H1NA	0.89 (2)	O4A—C11A	1.3440 (16)
N2A—C2A	1.3264 (18)	O4A—H1OA	0.88 (2)
N2A—H2NA	0.88 (2)	O5A—C13A	1.3213 (16)
N2A—H3NA	0.88 (2)	O5A—H2OA	0.86 (2)
C1A—C5A	1.359 (2)	O6A—C13A	1.2238 (17)
C1A—H1AA	0.9300	C7A—C8A	1.3840 (18)
C2A—C3A	1.4203 (19)	C7A—C12A	1.3977 (18)
C3A—C4A	1.366 (2)	C7A—H7AA	0.9300
C3A—H3AA	0.9300	C8A—C9A	1.3998 (18)
C4A—C5A	1.419 (2)	C9A—C10A	1.3801 (19)
C4A—H4AA	0.9300	C9A—H9AA	0.9300
C5A—C6A	1.502 (2)	C10A—C11A	1.4047 (18)
C6A—H6AA	0.9600	C10A—H10A	0.9300
C6A—H6AB	0.9600	C11A—C12A	1.4107 (18)
C6A—H6AC	0.9600	C12A—C13A	1.4791 (18)
N1B—C2B	1.3524 (18)	S1B—O1B	1.4513 (11)
N1B—C1B	1.3673 (18)	S1B—O3B	1.4558 (10)
N1B—H1NB	0.87 (2)	S1B—O2B	1.4737 (11)
N2B—C2B	1.3231 (18)	S1B—C8B	1.7651 (13)
N2B—H2NB	0.90 (2)	O4B—C11B	1.3485 (16)
N2B—H3NB	0.87 (2)	O4B—H1OB	0.86 (3)
C1B—C5B	1.3621 (19)	O5B—C13B	1.2275 (16)
C1B—H1BA	0.9300	O6B—C13B	1.3262 (16)
C2B—C3B	1.4229 (19)	O6B—H2OB	0.86 (2)
C3B—C4B	1.363 (2)	C7B—C8B	1.3833 (18)
C3B—H3BA	0.9300	C7B—C12B	1.4009 (18)
C4B—C5B	1.420 (2)	C7B—H7BA	0.9300
C4B—H4BA	0.9300	C8B—C9B	1.3969 (19)
C5B—C6B	1.506 (2)	C9B—C10B	1.3813 (19)
C6B—H6BA	0.9600	C9B—H9BA	0.9300
C6B—H6BB	0.9600	C10B—C11B	1.3994 (19)
C6B—H6BC	0.9600	C10B—H10B	0.9300
S1A—O2A	1.4545 (10)	C11B—C12B	1.4113 (18)
S1A—O3A	1.4580 (10)	C12B—C13B	1.4736 (18)
			
C2A—N1A—C1A	123.30 (12)	O3A—S1A—O1A	111.39 (6)
C2A—N1A—H1NA	121.4 (12)	O2A—S1A—C8A	106.31 (6)
C1A—N1A—H1NA	115.3 (12)	O3A—S1A—C8A	107.62 (6)
C2A—N2A—H2NA	119.6 (12)	O1A—S1A—C8A	105.57 (6)
C2A—N2A—H3NA	116.7 (12)	C11A—O4A—H1OA	107.3 (15)
H2NA—N2A—H3NA	122.3 (17)	C13A—O5A—H2OA	105.9 (15)
C5A—C1A—N1A	121.50 (13)	C8A—C7A—C12A	120.22 (12)
C5A—C1A—H1AA	119.2	C8A—C7A—H7AA	119.9
N1A—C1A—H1AA	119.2	C12A—C7A—H7AA	119.9
N2A—C2A—N1A	119.77 (13)	C7A—C8A—C9A	120.24 (12)
N2A—C2A—C3A	123.29 (13)	C7A—C8A—S1A	119.75 (10)
N1A—C2A—C3A	116.94 (12)	C9A—C8A—S1A	119.99 (10)
C4A—C3A—C2A	120.04 (13)	C10A—C9A—C8A	120.17 (12)
C4A—C3A—H3AA	120.0	C10A—C9A—H9AA	119.9
C2A—C3A—H3AA	120.0	C8A—C9A—H9AA	119.9
C3A—C4A—C5A	121.37 (13)	C9A—C10A—C11A	120.30 (12)
C3A—C4A—H4AA	119.3	C9A—C10A—H10A	119.8
C5A—C4A—H4AA	119.3	C11A—C10A—H10A	119.8
C1A—C5A—C4A	116.84 (13)	O4A—C11A—C10A	117.21 (12)
C1A—C5A—C6A	121.41 (14)	O4A—C11A—C12A	123.50 (12)
C4A—C5A—C6A	121.74 (13)	C10A—C11A—C12A	119.29 (12)
C5A—C6A—H6AA	109.5	C7A—C12A—C11A	119.64 (12)
C5A—C6A—H6AB	109.5	C7A—C12A—C13A	121.04 (12)
H6AA—C6A—H6AB	109.5	C11A—C12A—C13A	119.32 (12)
C5A—C6A—H6AC	109.5	O6A—C13A—O5A	123.14 (12)
H6AA—C6A—H6AC	109.5	O6A—C13A—C12A	122.18 (12)
H6AB—C6A—H6AC	109.5	O5A—C13A—C12A	114.67 (12)
C2B—N1B—C1B	123.07 (12)	O1B—S1B—O3B	113.74 (7)
C2B—N1B—H1NB	120.3 (14)	O1B—S1B—O2B	111.56 (7)
C1B—N1B—H1NB	116.6 (14)	O3B—S1B—O2B	111.44 (6)
C2B—N2B—H2NB	119.5 (14)	O1B—S1B—C8B	106.35 (6)
C2B—N2B—H3NB	118.2 (13)	O3B—S1B—C8B	107.66 (6)
H2NB—N2B—H3NB	121.9 (19)	O2B—S1B—C8B	105.54 (6)
C5B—C1B—N1B	121.57 (13)	C11B—O4B—H1OB	108.1 (17)
C5B—C1B—H1BA	119.2	C13B—O6B—H2OB	106.8 (15)
N1B—C1B—H1BA	119.2	C8B—C7B—C12B	120.05 (12)
N2B—C2B—N1B	119.93 (13)	C8B—C7B—H7BA	120.0
N2B—C2B—C3B	123.15 (13)	C12B—C7B—H7BA	120.0
N1B—C2B—C3B	116.91 (13)	C7B—C8B—C9B	120.24 (12)
C4B—C3B—C2B	120.18 (13)	C7B—C8B—S1B	119.28 (10)
C4B—C3B—H3BA	119.9	C9B—C8B—S1B	120.38 (10)
C2B—C3B—H3BA	119.9	C10B—C9B—C8B	120.42 (13)
C3B—C4B—C5B	121.44 (13)	C10B—C9B—H9BA	119.8
C3B—C4B—H4BA	119.3	C8B—C9B—H9BA	119.8
C5B—C4B—H4BA	119.3	C9B—C10B—C11B	120.15 (13)
C1B—C5B—C4B	116.81 (13)	C9B—C10B—H10B	119.9
C1B—C5B—C6B	121.38 (14)	C11B—C10B—H10B	119.9
C4B—C5B—C6B	121.80 (13)	O4B—C11B—C10B	117.80 (12)
C5B—C6B—H6BA	109.5	O4B—C11B—C12B	122.69 (12)
C5B—C6B—H6BB	109.5	C10B—C11B—C12B	119.50 (12)
H6BA—C6B—H6BB	109.5	C7B—C12B—C11B	119.63 (12)
C5B—C6B—H6BC	109.5	C7B—C12B—C13B	121.07 (12)
H6BA—C6B—H6BC	109.5	C11B—C12B—C13B	119.29 (12)
H6BB—C6B—H6BC	109.5	O5B—C13B—O6B	122.40 (12)
O2A—S1A—O3A	113.38 (6)	O5B—C13B—C12B	122.43 (12)
O2A—S1A—O1A	112.01 (6)	O6B—C13B—C12B	115.15 (11)
			
C2A—N1A—C1A—C5A	−0.6 (2)	C8A—C7A—C12A—C11A	1.5 (2)
C1A—N1A—C2A—N2A	−179.12 (13)	C8A—C7A—C12A—C13A	−178.13 (12)
C1A—N1A—C2A—C3A	0.21 (19)	O4A—C11A—C12A—C7A	176.05 (12)
N2A—C2A—C3A—C4A	179.36 (13)	C10A—C11A—C12A—C7A	−3.99 (19)
N1A—C2A—C3A—C4A	0.0 (2)	O4A—C11A—C12A—C13A	−4.4 (2)
C2A—C3A—C4A—C5A	0.0 (2)	C10A—C11A—C12A—C13A	175.62 (12)
N1A—C1A—C5A—C4A	0.6 (2)	C7A—C12A—C13A—O6A	−174.32 (13)
N1A—C1A—C5A—C6A	−179.37 (13)	C11A—C12A—C13A—O6A	6.1 (2)
C3A—C4A—C5A—C1A	−0.4 (2)	C7A—C12A—C13A—O5A	6.87 (19)
C3A—C4A—C5A—C6A	179.63 (14)	C11A—C12A—C13A—O5A	−172.73 (12)
C2B—N1B—C1B—C5B	−0.8 (2)	C12B—C7B—C8B—C9B	−0.2 (2)
C1B—N1B—C2B—N2B	−178.11 (13)	C12B—C7B—C8B—S1B	176.22 (10)
C1B—N1B—C2B—C3B	1.5 (2)	O1B—S1B—C8B—C7B	33.14 (13)
N2B—C2B—C3B—C4B	178.47 (14)	O3B—S1B—C8B—C7B	155.40 (11)
N1B—C2B—C3B—C4B	−1.2 (2)	O2B—S1B—C8B—C7B	−85.48 (12)
C2B—C3B—C4B—C5B	0.0 (2)	O1B—S1B—C8B—C9B	−150.46 (12)
N1B—C1B—C5B—C4B	−0.4 (2)	O3B—S1B—C8B—C9B	−28.20 (14)
N1B—C1B—C5B—C6B	−179.94 (13)	O2B—S1B—C8B—C9B	90.92 (13)
C3B—C4B—C5B—C1B	0.8 (2)	C7B—C8B—C9B—C10B	−0.1 (2)
C3B—C4B—C5B—C6B	−179.74 (14)	S1B—C8B—C9B—C10B	−176.49 (12)
C12A—C7A—C8A—C9A	1.5 (2)	C8B—C9B—C10B—C11B	−0.1 (2)
C12A—C7A—C8A—S1A	179.70 (10)	C9B—C10B—C11B—O4B	−179.52 (13)
O2A—S1A—C8A—C7A	21.40 (13)	C9B—C10B—C11B—C12B	0.6 (2)
O3A—S1A—C8A—C7A	143.18 (11)	C8B—C7B—C12B—C11B	0.7 (2)
O1A—S1A—C8A—C7A	−97.74 (11)	C8B—C7B—C12B—C13B	−178.08 (12)
O2A—S1A—C8A—C9A	−160.40 (11)	O4B—C11B—C12B—C7B	179.22 (12)
O3A—S1A—C8A—C9A	−38.61 (12)	C10B—C11B—C12B—C7B	−1.0 (2)
O1A—S1A—C8A—C9A	80.46 (12)	O4B—C11B—C12B—C13B	−1.9 (2)
C7A—C8A—C9A—C10A	−1.9 (2)	C10B—C11B—C12B—C13B	177.88 (13)
S1A—C8A—C9A—C10A	179.89 (11)	C7B—C12B—C13B—O5B	−178.04 (13)
C8A—C9A—C10A—C11A	−0.7 (2)	C11B—C12B—C13B—O5B	3.1 (2)
C9A—C10A—C11A—O4A	−176.43 (12)	C7B—C12B—C13B—O6B	3.28 (18)
C9A—C10A—C11A—C12A	3.6 (2)	C11B—C12B—C13B—O6B	−175.54 (12)

### Hydrogen-bond geometry (Å, °)

**Table:**

*D*—H···*A*	*D*—H	H···*A*	*D*···*A*	*D*—H···*A*
O4A—H1OA···O6A	0.88 (2)	1.84 (2)	2.6135 (14)	147 (2)
O4A—H1OA···O1B^i^	0.88 (2)	2.39 (2)	2.9581 (14)	123.2 (18)
O5A—H2OA···O2B^ii^	0.86 (2)	1.80 (2)	2.6609 (14)	172 (2)
O4B—H1OB···O5B	0.86 (3)	1.83 (2)	2.5918 (14)	147 (2)
O4B—H1OB···O2A^iii^	0.86 (3)	2.45 (2)	3.0349 (14)	125.4 (18)
O6B—H2OB···O1A	0.86 (2)	1.81 (2)	2.6664 (14)	178 (2)
N1A—H1NA···O3A^iv^	0.894 (19)	2.066 (19)	2.9057 (15)	156.0 (17)
N2A—H2NA···O2A^iv^	0.878 (19)	2.167 (19)	3.0043 (16)	159.1 (17)
N2A—H2NA···O5B^v^	0.878 (19)	2.417 (19)	2.8235 (16)	108.7 (13)
N2A—H3NA···O1A^v^	0.88 (2)	2.17 (2)	3.0472 (16)	175.6 (15)
N1B—H1NB···O3B^ii^	0.87 (2)	2.02 (2)	2.8547 (16)	161 (2)
N2B—H2NB···O1B^ii^	0.90 (2)	2.04 (2)	2.9188 (17)	166 (2)
N2B—H2NB···O6A^vi^	0.90 (2)	2.45 (2)	2.8254 (16)	105.9 (17)
N2B—H3NB···O2B^i^	0.87 (2)	2.26 (2)	3.1270 (17)	177.1 (18)
C7A—H7AA···O4B^iii^	0.93	2.58	3.4257 (16)	152
C7B—H7BA···O4A^i^	0.93	2.48	3.3116 (16)	148

Symmetry codes: (i) −*x*+1, −*y*+1, −*z*+1; (ii) *x*, *y*, *z*+1; (iii) −*x*+2, −*y*, −*z*+1; (iv) *x*, *y*+1, *z*; (v) −*x*+2, −*y*+1, −*z*+1; (vi) −*x*+1, −*y*+1, −*z*+2.
